# Effect of Preventive and Curative Fingolimod Treatment Regimens on Microglia Activation and Disease Progression in a Rat Model of Multiple Sclerosis

**DOI:** 10.1007/s11481-017-9741-x

**Published:** 2017-03-30

**Authors:** David Vállez García, Janine Doorduin, Daniele de Paula Faria, Rudi A. J. O. Dierckx, Erik F. J. de Vries

**Affiliations:** 1 Department of Nuclear Medicine and Molecular Imaging, University Medical Center Groningen, University of Groningen, Hanzeplein 1, PO Box 30.001, 9700 RB Groningen, The Netherlands; 2 Department of Radiology and Oncology, Faculty of Medicine, University of Sao Paulo, Sao Paulo, Brazil

**Keywords:** Fingolimod, Multiple sclerosis, Experimental autoimmune encephalomyelitis, Positron-emission tomography, Neuroinflammation

## Abstract

Fingolimod was the first oral drug approved for multiple sclerosis treatment. Its principal mechanism of action is blocking of lymphocyte trafficking. In addition, recent studies have shown its capability to diminish microglia activation. The effect of preventive and curative fingolimod treatment on the time-course of neuroinflammation was investigated in the experimental autoimmune encephalomyelitis rat model for multiple sclerosis. Neuroinflammatory progression was followed in Dark Agouti female rats after immunization. Positron-Emission tomography (PET) imaging with *(R)*-[^11^C]PK11195 was performed on day 11, 15, 19, 27, 29 and 34 during normal disease progression, preventive and curative treatments with fingolimod (1 mg/kg/day). Additionally, bodyweight and clinical symptoms were determined. Preventive treatment diminished bodyweight loss and inhibited the appearance of neurological symptoms. In non-treated rats, PET showed that neuroinflammation peaked in the brainstem at day 19, whereas the imaging signal was decreased in cortical regions. Both preventive and curative treatment reduced neuroinflammation in the brainstem at day 19. Eight days after treatment withdrawal, neuroinflammation had flared-up, especially in cortical regions. Preventive treatment with fingolimod suppressed clinical symptoms and neuroinflammation in the brainstem. After treatment withdrawal, clinical symptoms reappeared together with neuroinflammation in cortical regions, suggesting a different pathway of disease progression.

**Electronic supplementary material:**

The online version of this article (doi:10.1007/s11481-017-9741-x) contains supplementary material, which is available to authorized users.

## Introduction

Multiple sclerosis (MS) is an inflammatory and degenerative disease of the central nervous system characterized by demyelination. New therapies for MS have focused on suppression of relapses, and the reduction of lesions observed by magnetic resonance imaging (MRI). Fingolimod (FTY720; Gilenya®) was the first oral agent to be approved for treatment of MS. Results from clinical trials have consistently proved an effective decrease in relapse rates, new MRI lesions, disability progression and brain volume loss in patients with relapsing-remitting MS (Cohen et al. 2010; Kappos et al. 2010; Calabresi et al. 2014).

Fingolimod is a sphingosine-1-phosphate (S1P) analogue that is phosphorylated in vivo and binds as a functional antagonist to all the S1P receptor subtypes, except S1P_2_ (Brinkmann et al. 2002; Cohen and Chun 2011)_._ The S1P receptors are present on lymphocytes and play a key role in lymphocyte trafficking (Graeler and Goetzl 2002, 2004; Lo et al. 2005).

Fingolimod’s mechanism of action in MS is not known with certainty. However, fingolimod was shown to inhibit egress of lymphocytes from lymph nodes, thus preventing their recirculation and brain infiltration and the onset of an autoimmune reaction (Cohen and Chun 2011). Fingolimod can also penetrate the blood brain barrier (BBB) and bind to neurons, oligodendrocyte progenitor cells, astrocytes and microglia (Foster et al. 2007; Jeffery 2013). Fingolimod has recently been shown to suppress microglia activation in vitro (Coelho et al. 2007; Jackson et al. 2011; Noda et al. 2013; Cipriani et al. 2015) and in vivo in MS models (Anthony et al. 2014; Airas et al. 2015). This suggests that a combination of beneficial anti-inflammatory, neuroprotective and reparative effects may contribute to the efficacy of fingolimod in MS.

Microglia activation, a hallmark of neuroinflammation, is accompanied by increased expression of the 18-kDa translocator protein (TSPO), formerly known as the peripheral benzodiazepine receptor (Papadopoulos et al. 2006). This increase in TSPO expression can be visualized by positron-emission tomography (PET) with the tracer *(R)*-[^11^C]PK11195, as shown previously in animal models (Vowinckel et al. 1997; Abourbeh et al. 2012; Xie et al. 2012; Mattner et al. 2013; de Paula Faria et al. 2014a, b; Airas et al. 2015) and patients (Debruyne et al. 2003; Versijpt et al. 2005; Oh et al. 2011; Politis et al. 2012; Takano et al. 2013; Colasanti et al. 2014; Rissanen et al. 2014; Park et al. 2015; Giannetti et al. 2015).

The aim of this study was to evaluate how microglia activation is affected by fingolimod when the treatment was started before or after the appearance of the symptoms. To study this effect, repetitive *(R)*-[^11^C]PK11195 PET scans were performed in the experimental autoimmune encephalomyelitis (EAE) rat model.

## Material and Methods

### Animals

Adult female Dark Agouti rats (*n* = 28) at 8–10 weeks of age (153 ± 7 g) were obtained from Janvier (France). After arrival, the rats were allowed to acclimatize for at least 7 days. During the entire study, the rats were housed in pairs in Makrolon cages on a layer of wood shavings, in a room at constant temperature (21 ± 2 °C) and 12 h day/night periods. Commercial chow and water were available ad libitum.

All animal experiments were performed according to the Dutch Law on Animal Welfare and were approved by the Institutional Animal Care and Use Committee of the University of Groningen (DEC 6480B).

### Experimental Model

EAE was induced according to the previously described protocol (Ledeboer et al. 2003; de Paula Faria et al. 2014b). Briefly, rats were anesthetized with 5% isoflurane (mixed with medical air) and immunized intradermal, at both sides of the dorsal tail base, with 25 μg of endotoxin-free rat recombinant myelin oligodendrocyte glycoprotein_1–125_ (Tebu-bio) dissolved in 100 μL of 25 mM sodium acetate pH 4.0 and emulsified in 100 μL of Incomplete Freund’s Adjuvant (Difco Lab).

Bodyweight and clinical symptoms were evaluated daily by the same researcher. Symptoms were scored as: 0, no clinical symptoms; 0.5, limp distal tail; 1, completely limp tail; 2, ataxia; 3, moderate paraparesis; 3.5 unilateral hind-limb paralysis; 4, bilateral hind-limb paralysis; 5, bilateral hind-limb paralysis and bladder paralysis; and 6, moribund or dead. Water and powder food were provided by hand to those rats suffering paralysis. If needed, subcutaneous injections (1–2 times) of saline were given daily. Moribund rats with a score of 6 were terminated, and were included in the analysis with a score of 6 until the end of the experiment.

### Study Design

**Fig. 1:**
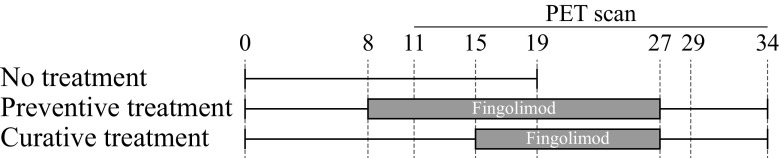
Study design. The rats were trained to receive oral gavage with water (5 ml/kg) from day 5 to 7. The non-treated group (*n* = 8) continued with administration of water from day 8 until day 19, when the rats where terminated. The preventive group (*n* = 10) received a treatment with fingolimod (1 mg/kg dissolved in 5 ml/kg of water) from day 8 until day 26, while the curative group (*n* = 10) received water from day 8 to 14, and the fingolimod treatment from day 15 until day 26. At day 27, the treatment was stopped for the preventive and curative group, and at day 34 the rats were terminated

The distribution of rats during the study is detailed in Table S1. Three groups were defined: a non-treated control group (*n* = 8), and a preventive (*n* = 10) and a curative treatment (*n* = 10) group. Fingolimod was administered daily to the treatment groups by orogastric gavage, which was shown to have no effect in corticosterone levels, body weight or food consumption (Turner et al. 2012). The rats were trained to receive the oral gavage with water (5 ml/kg) from day 5 to 7. The non-treated group received water instead of treatment until day 19, when these rats were terminated. The preventive treatment group received the drug from day 8 until day 26, while the curative treatment group was treated from day 15 (after the PET scan) until day 26. The treatment consisted of 1 mg/kg of fingolimod dissolved in 5 ml/kg of water. In both treatment groups, drug administration was stopped at day 27 and the rats were terminated after the last PET scan on day 34. A visual representation of the study design can be found in Fig. 1.

### PET Acquisition and Reconstruction

The synthesis of *(R)*-[^11^C]PK11195 was reported in detail elsewhere (Doorduin et al. 2009). Further details on the injected activity and mass can be found in Table 1. PET scans were performed with a microPET Focus 220 camera (Siemens Medical Solutions, USA) at day 11, 15, 19, 27, 29, and 34 after immunization. The first three scans were defined to monitor: initial symptoms (10–12 days), the first (14–15 days), and the second (19–20 days) relapse of symptoms, according to previous results (de Paula Faria et al. 2014b). The scan at day 27 was intended to reflect the neuroinflammatory situation at the end of treatment, and the last two scans (29 and 34 days) were defined to demonstrate the effects of treatment withdrawal.

**Table 1:** Injected dose and injected mass of *(R)*-[^11^C]PK11195

Scan	Group	Injected dose (MBq)			Injected mass (nmol)		
		Mean	±	SD	Mean	±	SD
day 11	No treatment	37	±	29	0.43	±	0.48
	Preventive treatment	58	±	15	0.53	±	0.41
	Curative treatment	59	±	18	0.33	±	0.11
day 15	No treatment	42	±	28	0.17	±	0.18
	Preventive treatment	47	±	19	0.57	±	0.61
	Curative treatment	32	±	14	0.67	±	0.49
day 19	No treatment	52	±	37	0.46	±	0.20
	Preventive treatment	54	±	31	0.86	±	0.61
	Curative treatment	39	±	25	0.52	±	0.26
day 27	Preventive treatment	33	±	19	0.52	±	0.48
	Curative treatment	38	±	24	0.45	±	0.45
day 29	Preventive treatment	46	±	17	0.36	±	0.16
	Curative treatment	48	±	15	0.36	±	0.15
day 34	Preventive treatment	54	±	9	0.53	±	0.31
	Curative treatment	53	±	6	0.65	±	0.28

Before each scan, the rats were anesthetized with 5% isoflurane mixed with oxygen (0.8 ml/min flow), and remained anesthetized with 1.5–2% isoflurane until the end of the scan. The tail vein was cannulated for tracer injection. The injected *(R)*-[^11^C]PK11195 dose was 46 ± 22 MBq, with an injected mass of 0.49 ± 0.39 nmol (no statistical differences between groups). About 40 min after tracer injection, the rats were placed into the camera in a prone position with the head in the field of view. At 45 min after tracer injection, a 30-min emission scan was started. A transmission scan of 515 s was obtained using a ^57^Co point source for attenuation and scatter correction.

List-mode data was reconstructed into a single frame (OSEM2D, 4 iterations and 16 subsets) after being normalized and corrected for attenuation, scatter and decay. Final images had a 256 × 256 × 95 matrix with a pixel width of 0.63 mm and a slice thickness of 0.79 mm. Standardized uptake value (SUV) images were constructed for all the scans, defined as: radioactivity concentration (MBq/cm^3^)/[injected dose (MBq)/body weight (g)].

### Image Registration

After reconstruction, PET images were processed with PMOD v3.6 (PMOD Technologies, Switzerland). A tracer-specific *(R)*-[^11^C]PK11195 template was constructed following the methodology described previously (Vállez García et al. 2015), using healthy female Dark Agouti rats (*n* = 12, weight 166 ± 11 g, 9–12 weeks old) (de Paula Faria et al. 2014b). The template was used as reference in the automatic rigid registration procedure. Volumes of interest (VOI) were defined based on previously constructed regions (Vállez García et al. 2015) for the right and left hemisphere, including the amygdala, brainstem, cerebellum, cortex, globus pallidum, hippocampus, hypothalamus, midbrain, septum, striatum and thalamus.

### Statistical Analysis

Statistical analysis was performed using IBM SPSS Statistics 20, and the results are presented as mean ± standard error (SE). The Generalized Estimating Equations (GEE) model (Hardin and Hilbe 2012) was used to account for the repeated measurements in the longitudinal design, and the missing data. The exchangeable correlation matrix was selected for the analysis, and the Wald test used to report *p*-values, which were considered statistically significant at *p* < 0.05 without correction for multiple comparisons.

#### Gain in Body Weight

The gain in bodyweight was calculated for each rat as the difference between the body weights minus the weight on the immunization day. For the statistical model, the ‘group’ and ‘day of the measurement’ were included as variables.

#### Clinical Symptoms

The clinical scores were analyzed including ‘group’ and the ‘day of the measurement’ as variables of interest in the model.

#### VOI-Based Analysis

An independent GEE model was created for each of the predefined VOIs, including the *(R)*-[^11^C]PK11195 uptake (SUV) as the outcome variable, and the variables ‘group’, ‘scan’, and its interaction as predictor terms in the model.

#### Voxel-Based Analysis

The SPM12 software (University College London, United Kingdom), in combination with SwE v1.2.2 (Guillaume et al. 2014) and SAMIT v1.2 (Vállez García et al. 2015), was used for the voxel-based analysis. PET images were smoothed using a 1.2 mm Gaussian kernel. Corrections for small sample size (‘type C2’) and estimation of the degrees of freedom (‘approximate III’) were included in the design. The variables ‘group’, ‘scan’, ‘clinical score’, and their interactions, were included in the statistical model. The level of significance was set to a high threshold of *p-uncorrected* = 0.02275 (*Z*-score = 2, α = 0.05, one-tailed hypothesis), and an extent threshold of 100 voxels (voxel size of 0.2 × 0.2 × 0.2 mm).

## Results

### Gain in Bodyweight

**Fig. 2:**
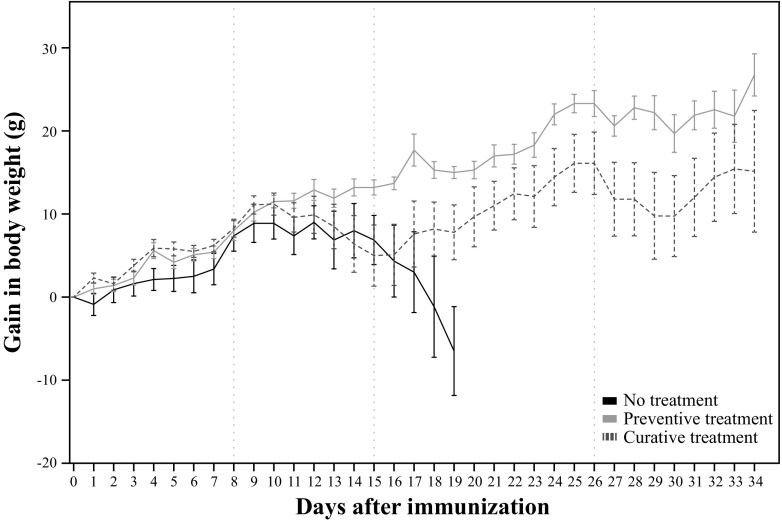
Gain in body weight over the study (mean ± SE). From left to right, the vertical dotted lines represent: the start of treatment in the preventive group (day 8), the start of treatment of curative group (day 15), and the last day of treatment (day 26)

A statistically significant difference on the gain in body weight was observed between groups (*p* = 0.009). In the pairwise group comparison, the ‘estimated marginal mean’ (*EMM*) on the gain in body weight of the curative treatment (8.2 ± 13.7 g) was not statistically different than the gain in the non-treated group (7.1 ± 13.4 g). However, the preventive treatment group showed an statistically significant higher gain of body weight (13.1 ± 12.6 g, *p* = 0.007) when compared with the non-treated group, and the curative group (*p* = 0.019) (Table 2; Fig. 2).

**Table 2:** Statistical comparison of the gain in bodyweight between groups, using the Generalized Estimating Equations model

	B	SE	95% Wald CI		*p*-value
			Lower	Upper	
(Intercept)	−0.38	1.96	−4.21	3.46	0.847
Preventive treatment	5.98	2.24	1.60	10.37	0.007
Curative treatment	1.11	2.48	−3.75	5.97	0.654
Day	0.50	0.09	0.33	0.66	<0.001

The parameter estimates were obtained using the no-treatment group as reference category

*B* Unstandardized coefficient; *SE* Standard error; *CI* Confidence interval

### Clinical Symptoms

**Fig. 3:**
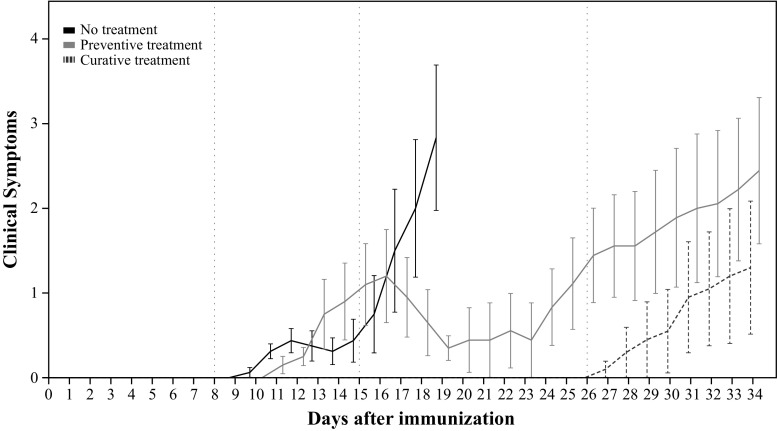
Clinical symptoms score (mean ± SE). From left to right the vertical lines represent: the start of treatment in the preventive group (day 8), the start of treatment of curative group (day 15), and the last day of treatment (day 26)

A statistically significant difference on the clinical score was found between groups (*p* < 0.001). In the pairwise comparison, the clinical score of the curative group (*EMM* = 0.7 ± 2.4) was not statistically different than the one found in the non-treated group (*EMM* = 0.7 ± 2.3). While the preventive treatment group showed a statistically significant lower clinical score (*EMM* = 0.1 ± 2.2) than the non-treated group (*p* < 0.001) and the curative treatment group (*p* = 0.021) (Table 3; Fig. 3).

**Table 3:** Statistical comparison of the clinical symptoms, using the Generalized Estimating Equations model

	B	SE	95% Wald CI		*p*-value
			Lower	Upper	
(Intercept)	−0.07	0.15	−0.37	0.22	0.635
Preventive treatment	−0.59	0.15	−0.89	−0.30	<0.001
Curative treatment	0.03	0.21	−0.38	0.43	0.895
Day	0.05	0.01	0.02	0.08	0.001

The parameter estimates were obtained using the saline group as reference category

*B* Unstandardized coefficient; *SE* Standard error; *CI* Confidence interval

### VOI-Based Analysis

**Fig. 4:**
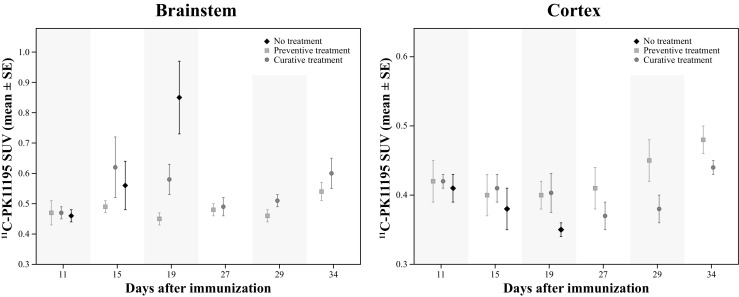
*(R)*-[^11^C]PK11195 PET uptake (mean ± SE) in the brainstem (*left*) and in the cerebral cortex (*right*). Both regions revealed a statistically significant difference between groups in the overall analysis

VOI-based analysis of *(R)*-[^11^C]PK11195 PET scans revealed a statistically significant difference between groups in the brainstem (*p* = 0.024) and cortical (*p* = 0.030) regions. No other overall differences were found between groups in any of the other brain regions. When explored in more detail, a statistically significant lower tracer uptake was found in the brainstem in the preventive treatment group (*EMM* = 0.48 ± 0.01, *p* = 0.023), but not in the curative treatment group (*EMM* = 0.53 ± 0.03), as compared with the uptake in the non-treated group (*EMM* = 0.62 ± 0.06). In addition, a statistically significant higher *(R)*-[^11^C]PK11195 uptake was found in the cortex of the preventive treatment group (*EMM* = 0.43 ± 0.01, *p* = 0.004) when compared with the non-treated group (*EMM* = 0.38 ± 0.01). However, no statistical difference with the other groups was observed for the curative treatment group (*EMM* = 0.40 ± 0.01) in the cortical region. Based on these results, a more detailed exploration of the data was performed for the brainstem and cortex region (Table 4, and Fig. 4).

**Table 4:** *(R)*-[^11^C]PK11195 PET uptake in the brainstem and cortex. These regions showed an overall statistically significant difference between groups (*p* < 0.05) in the Generalized Estimating Equations model

		Day 11			Day 15			Day 19			Day 27			Day 29			Day 34		
		Mean	±	SE	Mean	±	SE	Mean	±	SE	Mean	±	SE	Mean	±	SE	Mean	±	SE
Brainstem	No treatment	0.46	±	0.02	0.55	±	0.08	0.85	±	0.12									
	Preventive	0.48	±	0.04	0.52	±	0.01	0.45	±	0.02	0.48	±	0.02	0.47	±	0.02	0.50	±	0.01
	Curative	0.46	±	0.02	0.59	±	0.09	0.58	±	0.05	0.49	±	0.03	0.50	±	0.02	0.60	±	0.06
Cortex	No treatment	0.41	±	0.02	0.38	±	0.02	0.35	±	0.01									
	Preventive	0.42	±	0.03	0.42	±	0.02	0.40	±	0.02	0.41	±	0.03	0.45	±	0.02	0.46	±	0.02
	Curative	0.42	±	0.01	0.41	±	0.02	0.40	±	0.02	0.37	±	0.02	0.38	±	0.02	0.44	±	0.01

Only those regions with statistical significant differences are included

*SE* Standard error

#### Brainstem Region

In the brainstem region (Fig. 4, left), the within-group exploration of the VOI-based results showed that the non-treated group had a statistically significant increase of *(R)*-[^11^C]PK11195 uptake on day 19 as compared to day 15 (*p* = 0.002) and day 11 (*p* = 0.001). Moreover, the preventive treatment group showed a statistically significant higher uptake at day 34, when compared to day 19 (*p* = 0.018), at day 15 when compared to day 19 (*p* < 0.001) and day 29 (*p* = 0.041). Finally, the curative treatment group showed a higher uptake at day 19 (*p* = 0.006), day 29 (*p* = 0.028), and day 34 (*p* = 0.033) when compared to day 11; and a higher uptake at day 34 than at day 27 (*p* = 0.017).

In addition, in a between-groups comparison, only statistically significant differences were observed between the different treatment regimens on day 19. Preventive treatment resulted in lower tracer uptake than the curative treatment (*p* = 0.017). And both, the preventive and curative treatment groups, exhibited a lower tracer uptake in the brainstem than the non-treated group (*p* = 0.001 and *p* = 0.036 respectively).

#### Cortical Region

In the cortical region (Fig. 4, right), for the within-group comparison, a statistically significant decrease in the *(R)*-[^11^C]PK11195 uptake was observed in the non-treated group at day 19 (*p* = 0.029) when compared to day 11. In the preventive treatment group, an increased uptake was found at day 34 when compared to day 15 (*p* = 0.037) and day 19 (*p* = 0.041). In the curative treatment group, a decrease in the uptake was found at day 27 (*p* = 0.033) and day 29 (*p* = 0.024) when compared to day 11. In addition, an increased uptake was observed in the curative group at day 34 when compared to day 19 (*p* = 0.043), day 27 (*p* = 0.002), and day 29 (*p* < 0.001).

Moreover, between-group comparison revealed a significantly lower uptake in the non-treated group at day 19 as compared with preventive (*p* = 0.032), but not with the curative treated rats (*p* = 0.070). Additionally, a statistically significant higher uptake was also found at day 29 in the preventive group compared with the curative treatment group (*p* = 0.014).

### Voxel-Based Analysis

**Fig. 5:**
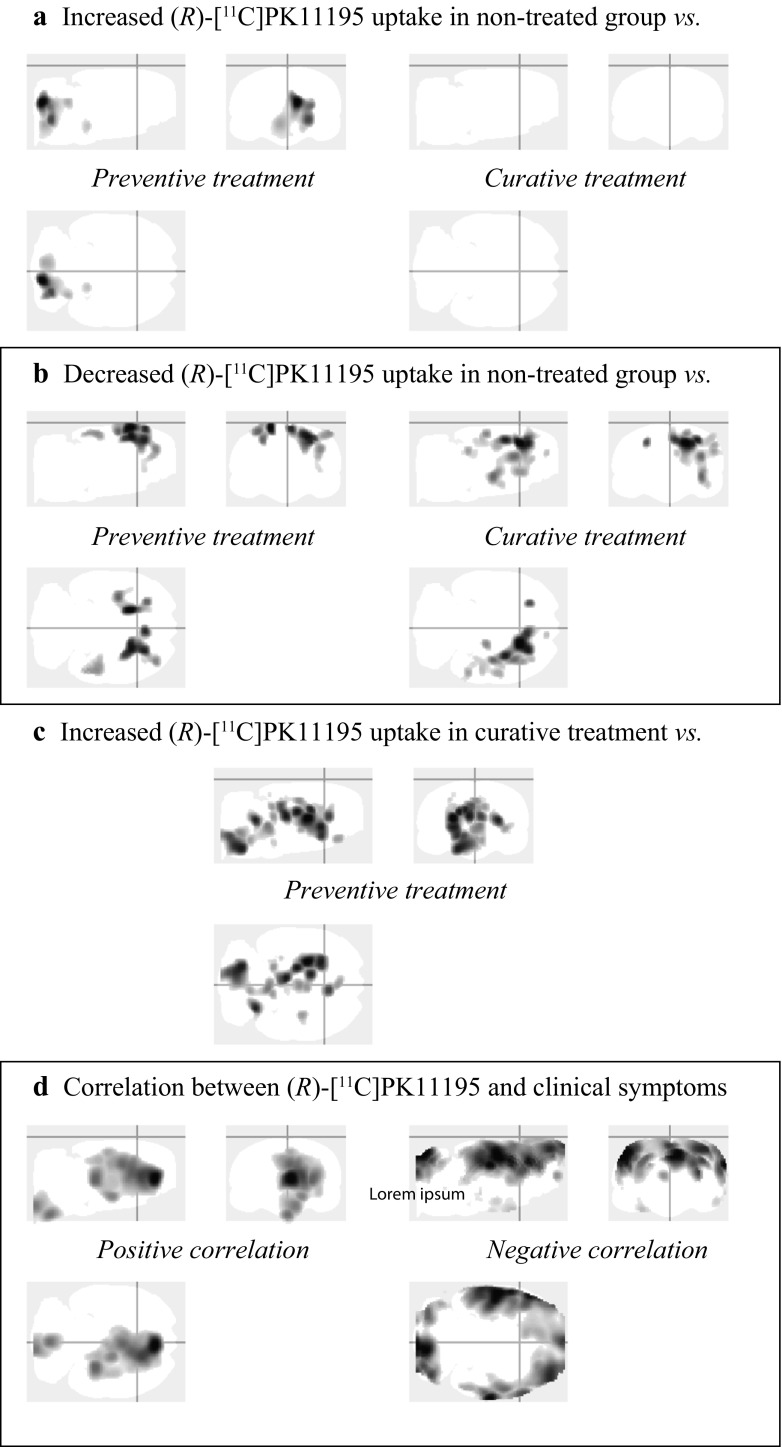
Maximum intensity projection (i.e. ‘glass-brain’ display) of the voxel-based analysis. At day 19, regions with a statistically significant **a** increase and **b** decrease of *(R)*-[^11^C]PK11195 uptake in the non-treated group when compared with the preventive (*left panels*) and curative (*right panels*) treatment groups. **c** At day 19, regions with a statistically significant increase of *(R)*-[^11^C]PK11195 uptake in the curative treatment group when compared with the preventive group. **d** Correlation of the clinical score with the *(R)*-[^11^C]PK11195 PET uptake, including all time points and groups

Exploration of the results by voxel-based analysis (Fig. 5; Table S2) was restricted to the group comparisons at day 19 (based on previous results of the VOI-based analysis) and to the correlation between the clinical scores and *(R)*-[^11^C]PK11195 uptake.

#### Day 19

Statically significant increased uptake was found mostly in the brainstem and cerebellum of the non-treated group when compared to the preventive treatment group, but not when compared to curative treatment group. At the same time, a decreased *(R)*-[^11^C]PK11195 uptake was found in in the cortical region and the striatum of the non-treated group when compared to the preventive and the curative treatment groups. When both treatment groups were compared, an increased *(R)*-[^11^C]PK11195 uptake was found, mostly located in the brainstem, midbrain, striatum, and thalamus of the curative treatment group as compared to the preventive treatment group.

#### Clinical Symptoms

The clinical score showed a strong statistical correlation with the *(R)*-[^11^C]PK11195 uptake, with a positive correlation in the brainstem, cortex, hippocampus, septum, striatum, and thalamus; and a negative correlation in most of the cortex and cerebellum.

## Discussion

In the present study, EAE rats were monitored for disease progression and fingolimod treatment effects. Differences in the bodyweight gain were observed between groups, with a significant decrease in bodyweight in those animals presenting clinical symptoms. This effect can be explained by inflammatory-induced cachexia, a common process in all the EAE models, an effect observed also previously by our group (de Paula Faria et al. 2014b). In this respect, fingolimod appears to improve the animals’ wellbeing, supporting the evidence of an immune-modulatory action of the drug, e.g. by preventing lymphocytes to exit the lymph nodes and thus to infiltrate into the brain (Cohen and Chun 2011), and by suppressing microglia activation via p38 mitogen-activated protein kinases (Cipriani et al. 2015).

The brainstem seems to be the most affected brain region in the EAE rat model, with a progressive increase in *(R)*-[^11^C]PK11195 uptake (neuroinflammation) over time. These results are in agreement with those published previously by de Paula Faria et al. (de Paula et al. 2014b), where the increased *(R)*-[^11^C]PK11195 uptake in the brainstem was confirmed by Iba1 immunohistochemistry, demonstrating the presence of activated microglia and/or macrophages in this region. The treatment with fingolimod resulted in a clear decrease in the neuroinflammation in the brainstem, as demonstrated by a reduction in the *(R)*-[^11^C]PK11195 uptake detected with the PET camera. In those rats where the treatment was started before the onset of the symptoms (preventive treatment) the benefits of fingolimod were superior to those obtained in rats where the drug was administered when the initial symptoms were already present.

In the present study, the neuroinflammatory process was clearly manifest in the non-treated animals at day 19. This inflammatory process was significantly reduced with the administration of fingolimod (1 mg/kg per day). This effect was significantly stronger in the preventive treatment group, in which fingolimod was administered before the appearance of the first clinical symptoms, than in the curative treatment group, where the administration of the drug was delayed until the first relapse of the symptoms at day 15. Moreover, no evidence of the disease was detected in the animals following the preventive regimen during the whole period in which the animals were receiving the drug, neither according to the *(R)*-[^11^C]PK11195 uptake detected by the PET camera nor according to the manifestation of clinical symptoms related with EAE disease progression. In the curative treatment group, on the other hand, the drug could partly reduce the existing symptoms, but was not capable to completely cure all symptoms that were already present. Consequently, some symptoms remained present for the rest of the study. In line with this observation, curative administration of the drug fingolimod partially extinguished the initial neuroinflammatory process in the brainstem that had started during the period before fingolimod treatment (from day 8 to 15). The statistically significant difference in tracer uptake between the preventive and the curative treatment group at day 19 was not present anymore at day 27 (end of treatment), as the levels of *(R)*-[^11^C]PK11195 uptake in the brainstem of curatively treated animals went back to those detected during the first scan on day 11. These data suggest that fingolimod needs few days to quench neuroinflammation and thus delay disease progression, but it could not completely reverse already existing damage.

Interestingly, clinical symptoms seem to positively correlate with *(R)*-[^11^C]PK11195 uptake in the brainstem, globus pallidum, hippocampus, thalamus and septum. A negative correlation between clinical symptoms and *(R)*-[^11^C]PK11195 uptake was also observed in part of the cortex and cerebellum. The positive correlation between *(R)*-[^11^C]PK11195 uptake in several brain regions and the clinical score likely reflects the ongoing neuroinflammatory process caused by the EAE disease progression, while the negative correlation may be explained by the migration of microglia from cortical regions to sites of neuroinflammation in the brainstem and midbrain. This migration of microglia seems to be reflected at day 19 in the statistical lower *(R)*-[^11^C]PK11195 signal observed in the cortical region of non-treated group, when compared with the preventive and the curative treatments, a result observed both in the voxel-based and in the VOI-based analysis. This migratory hypothesis, however, requires further investigation.

Preventive treatment with fingolimod suppressed the manifestation of clinical symptoms and neuroinflammation in the brainstem, while curative treatment was able to partly reduce the consequences of EAE. After removal of the treatment, clinical symptoms started to (re)appear, but these symptoms were less severe than those observed in the non-treated group (day 11–19). This process was accompanied by neuroinflammation, not only in the brainstem but also in cortical regions. These results are consistent with lymphocytes leaving the lymph nodes and returning to circulation. Interestingly, the cortical neuroinflammation was not observed in non-treated EAE rats, suggesting a different disease progression pattern after withdrawal of fingolimod treatment. However, it remains unclear how fingolimod could have affected the neuroinflammation pattern after withdrawal of the drug.

In summary, preventive treatment with fingolimod is able to suppress the manifestation of clinical symptoms and the associated neuroinflammatory process that takes place in the brainstem of EAE rats, while the curative treatment could reduce neuroinflammation and moderate, but not fully reverse, the symptoms in this MS model. After removal of the treatment, the clinical symptoms started to (re)appear and were accompanied by neuroinflammation in cortical regions, suggesting a different mode of disease progression after withdrawal from fingolimod treatment.

## Electronic supplementary material

Table S1 (DOCX 14 kb)

Table S2 (DOCX 14 kb)
