# Supplementary material for: Effect of Preventive and Curative Fingolimod Treatment Regimens on Microglia Activation and Disease Progression in a Rat Model of Multiple Sclerosis
Source: J Neuroimmune Pharmacol. 2017 Mar 30;12(3):521–30. doi: 10.1007/s11481-017-9741-x (PMC5527053; doi:10.1007/s11481-017-9741-x)
Supplement: Supplementary file 1 — (DOCX 14 kb) [file 11481_2017_9741_MOESM1_ESM.docx]

| Table S1. Distribution of rats during the study   \|  \| Animal \| day 11 \| day 15 \| day 19 \| day 27 \| day 29 \| day 34 \| \| --- \| --- \| --- \| --- \| --- \| --- \| --- \| --- \| \| Saline \| 3 \| Yes \| † \| † \|  \|  \|  \| \|  \| 4 \| Yes \| No \| Yes \|  \|  \|  \| \|  \| 7 \| Yes \| † \| † \|  \|  \|  \| \|  \| 8 \| Yes \| Yes \| Yes \|  \|  \|  \| \|  \| 9 \| Yes \| Yes \| Yes \|  \|  \|  \| \|  \| 10 \| Yes \| Yes \| Yes \|  \|  \|  \| \|  \| 11 \| Yes \| Yes \| Yes \|  \|  \|  \| \|  \| 12 \| No \| Yes \| Yes \|  \|  \|  \| \|  \| *Total:* \| *7* \| *5* \| *6* \|  \|  \|  \| \| Preventive treatment \| 1 \| No \| No \| Yes \| Yes \| No \| Yes \| \|  \| 2 \| Yes \| No \| Yes \| No \| Yes \| † \| \|  \| 13 \| No \| Yes \| Yes \| Yes \| Yes \| † \| \|  \| 14 \| Yes \| Yes \| Yes \| Yes \| Yes \| Yes \| \|  \| 18 \| No \| No \| Yes \| Yes \| Yes \| Yes \| \|  \| 19 \| No \| No \| Yes \| Yes \| Yes \| Yes \| \|  \| 21 \| Yes \| No \| Yes \| Yes \| Yes \| Yes \| \|  \| 22 \| Yes \| No \| Yes \| Yes \| Yes \| Yes \| \|  \| 23 \| Yes \| Yes \| Yes \| Yes \| Yes \| Yes \| \|  \| 24 \| Yes \| Yes \| Yes \| Yes \| Yes \| Yes \| \|  \| *Total:* \| *6* \| *4* \| *10* \| *9* \| *9* \| *8* \| \| Curative treatment \| 5 \| Yes \| No \| Yes \| Yes \| No \| † \| \|  \| 6 \| Yes \| No \| † \| † \| † \| † \| \|  \| 15 \| No \| Yes \| Yes \| Yes \| Yes \| Yes \| \|  \| 16 \| Yes \| Yes \| Yes \| Yes \| Yes \| † \| \|  \| 17 \| No \| No \| Yes \| Yes \| Yes \| Yes \| \|  \| 20 \| No \| No \| Yes \| No \| Yes \| Yes \| \|  \| 25 \| Yes \| No \| Yes \| Yes \| Yes \| Yes \| \|  \| 26 \| Yes \| No \| Yes \| Yes \| Yes \| Yes \| \|  \| 27 \| Yes \| Yes \| Yes \| Yes \| Yes \| Yes \| \|  \| 28 \| Yes \| Yes \| Yes \| Yes \| Yes \| † \| \|  \| *Total:* \| *7* \| *4* \| *9* \| *8* \| *8* \| *6* \|   Yes: Successful scan; No: Error during scan; † Animal died |
| --- | --- | --- | --- | --- | --- | --- | --- | --- | --- | --- | --- | --- | --- | --- | --- | --- | --- | --- | --- | --- | --- | --- | --- | --- | --- | --- | --- | --- | --- | --- | --- | --- | --- | --- | --- | --- | --- | --- | --- | --- | --- | --- | --- | --- | --- | --- | --- | --- | --- | --- | --- | --- | --- | --- | --- | --- | --- | --- | --- | --- | --- | --- | --- | --- | --- | --- | --- | --- | --- | --- | --- | --- | --- | --- | --- | --- | --- | --- | --- | --- | --- | --- | --- | --- | --- | --- | --- | --- | --- | --- | --- | --- | --- | --- | --- | --- | --- | --- | --- | --- | --- | --- | --- | --- | --- | --- | --- | --- | --- | --- | --- | --- | --- | --- | --- | --- | --- | --- | --- | --- | --- | --- | --- | --- | --- | --- | --- | --- | --- | --- | --- | --- | --- | --- | --- | --- | --- | --- | --- | --- | --- | --- | --- | --- | --- | --- | --- | --- | --- | --- | --- | --- | --- | --- | --- | --- | --- | --- | --- | --- | --- | --- | --- | --- | --- | --- | --- | --- | --- | --- | --- | --- | --- | --- | --- | --- | --- | --- | --- | --- | --- | --- | --- | --- | --- | --- | --- | --- | --- | --- | --- | --- | --- | --- | --- | --- | --- | --- | --- | --- | --- | --- | --- | --- | --- | --- | --- | --- | --- | --- | --- | --- | --- | --- | --- | --- | --- | --- | --- | --- | --- | --- | --- | --- | --- | --- | --- | --- | --- | --- | --- | --- | --- | --- | --- | --- | --- | --- | --- | --- | --- | --- | --- | --- | --- | --- | --- | --- | --- | --- | --- | --- | --- | --- | --- | --- |
