# Supplementary material for: Effect of Preventive and Curative Fingolimod Treatment Regimens on Microglia Activation and Disease Progression in a Rat Model of Multiple Sclerosis
Source: J Neuroimmune Pharmacol. 2017 Mar 30;12(3):521–30. doi: 10.1007/s11481-017-9741-x (PMC5527053; doi:10.1007/s11481-017-9741-x)
Supplement: Supplementary file 2 — (DOCX 14 kb) [file 11481_2017_9741_MOESM2_ESM.docx]

| Table S2. Results of the voxel-based analysis of neuroinflammation, by means of *(R)*-[^11^C]PK11195, represented by brain regions   \| **Contrast** \| **Brain Region** \| **Voxels** \| **Z-score** \| **±** \| **SD** \| \| --- \| --- \| --- \| --- \| --- \| --- \| \| No treatment > Preventive \| Brainstem \| 1141 \| 2.08 \| ± \| 0.07 \| \|  \| Cerebellum \| 2431 \| 2.14 \| ± \| 0.13 \| \|  \| Midbrain \| 50 \| 2.05 \| ± \| 0.03 \| \|  \|  \|  \|  \|  \|  \| \| No treatment < Preventive \| Cortex \| 3234 \| 2.20 \| ± \| 0.15 \| \|  \| Striatum \| 174 \| 2.13 \| ± \| 0.12 \| \|  \|  \|  \|  \|  \|  \| \| No treatment < Curative \| Amygdala \| 188 \| 2.15 \| ± \| 0.11 \| \|  \| Cortex \| 2273 \| 2.20 \| ± \| 0.15 \| \|  \| Globus Pallidum \| 71 \| 2.17 \| ± \| 0.11 \| \|  \| Hippocampus \| 176 \| 2.07 \| ± \| 0.06 \| \|  \| Striatum \| 1792 \| 2.18 \| ± \| 0.16 \| \|  \|  \|  \|  \|  \|  \| \| Preventive < Curative \| Brainstem \| 2839 \| 2.21 \| ± \| 0.15 \| \|  \| Cerebellum \| 474 \| 2.13 \| ± \| 0.10 \| \|  \| Cortex \| 35 \| 2.07 \| ± \| 0.05 \| \|  \| Globus Pallidum \| 410 \| 2.21 \| ± \| 0.09 \| \|  \| Hippocampus \| 478 \| 2.13 \| ± \| 0.09 \| \|  \| Midbrain \| 1050 \| 2.14 \| ± \| 0.10 \| \|  \| Septum \| 301 \| 2.16 \| ± \| 0.09 \| \|  \| Striatum \| 1085 \| 2.08 \| ± \| 0.07 \| \|  \| Thalamus \| 1172 \| 2.27 \| ± \| 0.17 \| \|  \|  \|  \|  \|  \|  \| \| Clinical Score (Positive) \| Amygdala \| 46 \| 2.11 \| ± \| 0.10 \| \|  \| Brainstem \| 2294 \| 2.26 \| ± \| 0.16 \| \|  \| Cortex \| 1478 \| 2.40 \| ± \| 0.38 \| \|  \| Globus Pallidum \| 356 \| 2.16 \| ± \| 0.10 \| \|  \| Hippocampus \| 2929 \| 2.24 \| ± \| 0.17 \| \|  \| Hypothalamus \| 473 \| 2.23 \| ± \| 0.17 \| \|  \| Midbrain \| 450 \| 2.17 \| ± \| 0.15 \| \|  \| Septum \| 1679 \| 2.50 \| ± \| 0.22 \| \|  \| Striatum \| 3767 \| 2.42 \| ± \| 0.28 \| \|  \| Thalamus \| 2999 \| 2.23 \| ± \| 0.17 \| \|  \|  \|  \|  \|  \|  \| \| Clinical Score (Negative) \| Amygdala \| 60 \| 2.09 \| ± \| 0.07 \| \|  \| Cerebellum \| 3118 \| 2.52 \| ± \| 0.40 \| \|  \| Cortex \| 12301 \| 2.23 \| ± \| 0.18 \| |
| --- | --- | --- | --- | --- | --- | --- | --- | --- | --- | --- | --- | --- | --- | --- | --- | --- | --- | --- | --- | --- | --- | --- | --- | --- | --- | --- | --- | --- | --- | --- | --- | --- | --- | --- | --- | --- | --- | --- | --- | --- | --- | --- | --- | --- | --- | --- | --- | --- | --- | --- | --- | --- | --- | --- | --- | --- | --- | --- | --- | --- | --- | --- | --- | --- | --- | --- | --- | --- | --- | --- | --- | --- | --- | --- | --- | --- | --- | --- | --- | --- | --- | --- | --- | --- | --- | --- | --- | --- | --- | --- | --- | --- | --- | --- | --- | --- | --- | --- | --- | --- | --- | --- | --- | --- | --- | --- | --- | --- | --- | --- | --- | --- | --- | --- | --- | --- | --- | --- | --- | --- | --- | --- | --- | --- | --- | --- | --- | --- | --- | --- | --- | --- | --- | --- | --- | --- | --- | --- | --- | --- | --- | --- | --- | --- | --- | --- | --- | --- | --- | --- | --- | --- | --- | --- | --- | --- | --- | --- | --- | --- | --- | --- | --- | --- | --- | --- | --- | --- | --- | --- | --- | --- | --- | --- | --- | --- | --- | --- | --- | --- | --- | --- | --- | --- | --- | --- | --- | --- | --- | --- | --- | --- | --- | --- | --- | --- | --- | --- | --- | --- | --- | --- | --- | --- | --- | --- | --- | --- | --- | --- | --- | --- | --- | --- | --- | --- | --- | --- | --- | --- | --- | --- | --- | --- | --- | --- | --- | --- |
